# Protein Nanotubes: From Bionanotech towards Medical Applications

**DOI:** 10.3390/biomedicines7020046

**Published:** 2019-06-23

**Authors:** Gerald F. Audette, Ayat Yaseen, Nicholas Bragagnolo, Raj Bawa

**Affiliations:** 1 Department of Chemistry and the Centre for Research on Biomolecular Interactions, York University, Toronto, ON M3J 1P3, Canada; yaseen.ayat@outlook.com (A.Y.); nickb13@my.yorku.ca (N.B.); 2 Patent Law Department, Bawa Biotech LLC, Ashburn, VA 20147, USA; 3 Guanine Inc., Rensselaer, NY 12144-3463, USA; 4 Pharmaceutical Research Institute of Albany College of Pharmacy and Health Sciences, Albany, NY 12208, USA

**Keywords:** nanobiotechnology, protein nanotubes (PNTs), protein engineering, self-assembly, nanowires, drug delivery, imaging agents, biosensors

## Abstract

Nanobiotechnology involves the study of structures found in nature to construct nanodevices for biological and medical applications with the ultimate goal of commercialization. Within a cell most biochemical processes are driven by proteins and associated macromolecular complexes. Evolution has optimized these protein-based nanosystems within living organisms over millions of years. Among these are flagellin and pilin-based systems from bacteria, viral-based capsids, and eukaryotic microtubules and amyloids. While carbon nanotubes (CNTs), and protein/peptide-CNT composites, remain one of the most researched nanosystems due to their electrical and mechanical properties, there are many concerns regarding CNT toxicity and biodegradability. Therefore, proteins have emerged as useful biotemplates for nanomaterials due to their assembly under physiologically relevant conditions and ease of manipulation via protein engineering. This review aims to highlight some of the current research employing protein nanotubes (PNTs) for the development of molecular imaging biosensors, conducting wires for microelectronics, fuel cells, and drug delivery systems. The translational potential of PNTs is highlighted.

## 1. Introduction

The term bionanotechnology refers to the use of biological molecules engineered to form nanoscale building materials. The assembly of small molecules into more complex higher ordered structures is referred to as the “bottom-up” process, in contrast to nanotechnology which typically uses the “top-down” approach of producing smaller macroscale devices. These biological molecules include DNA, lipids, peptides, and more recently, proteins. The intrinsic ability of nucleic acid bases to bind to one another due to their complementary sequence allows for the creation of useful materials. It is no surprise that they were one of the first biological molecules to be implemented for nanotechnology [1,2,3,4]. Similarly, the unique amphiphilicity of lipids and their diversity of head and tail chemistries provide a powerful outlet for nanotechnology [5]. Peptides are also emerging as intriguing and versatile drug delivery systems (recently reviewed in [6]), with secondary and tertiary structure induced upon self-assembly. This rapidly evolving field is now beginning to explore how whole proteins can be utilized as nanoscale drug delivery systems [7]. The organized quaternary assembly of proteins as nanofibers and nanotubes is being studied as biological scaffolds for numerous applications. These applications include tissue engineering, chromophore and drug delivery, wires for bio-inspired nano/microelectronics, and the development of biosensors.

The molecular self-assembly observed in protein-based systems is mediated by non-covalent interactions such as hydrogen bonds, electrostatic, hydrophobic and van der Waals interactions. When taken on a singular level these bonds are relatively weak, however combined as a whole they are responsible for the diversity and stability observed in many biological systems. Proteins are amphipathic macromolecules containing both non-polar (hydrophobic) and polar (hydrophilic) amino acids which govern protein folding. The hydrophilic regions are exposed to the solvent and the hydrophobic regions are oriented within the interior forming a semi-enclosed environment. The 20 naturally occurring amino acids used as building blocks for the production of proteins have unique chemical characteristics allowing for complex interactions such as macromolecular recognition and the specific catalytic activity of enzymes. These properties make proteins particularly attractive for the development of biosensors, as they are able to detect disease-associated analytes in vivo and carry out the desired response. Furthermore, the use of protein nanotubes (PNTs) for biomedical applications is of particular interest due to their well-defined structures, assembly under physiologically relevant conditions, and manipulation through protein engineering approaches [8]; such properties of proteins are difficult to achieve with carbon or inorganically derived nanotubes. For these reasons, groups are studying the immobilization of peptides and proteins onto carbon nanotubes (CNTs) in order to enhance several properties of biocatalysis such as thermal stability, pH, operating conditions etc. of the immobilized proteins/enzymes for applications in bionanotechnology and bionanomedicine. The effectiveness of immobilization is dependent on the targeted outcome, whether it is toward high sensitivity, selectivity or short response time and reproducibility [9]. A classic example of this is the glucose biosensor [10,11], where glucose oxidase (GOx) is immobilized onto CNTs, for detection of blood glucose levels; this approach can also be adapted for the development of GOx-CNT based biocatalysis for micro/nanofuel cells for wearable/implantable devices [9,12,13,14].

The use of proteins for the de novo production of nanotubes continues to prove quite challenging given the increased complexity that comes with fully folded tertiary structures. As a result, many groups have looked to systems found in nature as a starting point for the development of biological nanostructures. Two of these systems are found in bacteria, which produce fiber-like protein polymers allowing for the formation of extended flagella and pili. These naturally occurring structures consist of repeating monomers forming helical filaments extending from the bacterial cell wall with roles in intra and inter-cellular signaling, energy production, growth, and motility [15]. Another natural system of interest has been the adaptation of viral coat proteins for the production of nanowires and targeted drug delivery. The artificial modification of multimer ring proteins such as wild-type *trp* tRNA-binding attenuating protein (TRAP) [16,17,18], *P. aeruginosa* Hcp1 [19], stable protein 1 (SP1) [20], and the propanediol-utilization microcompartment shell protein PduA [21], have successfully produced nanotubes with modified dimensions and desired chemical properties. We discuss recent advances made in using protein nanofibers and self-assembling PNTs for a variety of applications.

## 2. Protein Nanofibers and Nanotubes (NTs) from Bacterial Systems

Progress in our understanding of both protein structure and function making up natural nanosystems allows us to take advantage of their potential in the fields of bionanotechnology and nanomedicine. Understanding how these systems self-assemble, how they can be modified through protein engineering, and exploring ways to produce nanotubes in vitro is of critical importance for the development of novel synthetic materials.

### 2.1. Flagella-Based Protein Nanofibers and Nanotubes

Flagella are hair-like structures produced by bacteria made up of three general components: a membrane bound protein gradient-driven pump, a joint hook structure, and a long helical fiber. The repeating unit of the long helical fiber is the FliC (flagellin) protein and is employed primarily for cellular motility. These fibers usually vary in length between 10–15 µm with an outer diameter of 12–25 nm and an inner diameter of 2–3 nm. Flagellin is a globular protein composed of four distinct domains: D0, D1, D2, and D3 [22]. The D0, D1 and part of the D2 domain are required for self-assembly into fibers and are largely conserved, while regions of the D2 domain and the entire D3 domain are highly variable [23,24], making them available for point mutations or insertion of loop peptides. The ability to display well-defined functional groups on the surface of the flagellin protein makes it an attractive model for the generation of ordered nanotubes. Up to 30,000 monomers of the FliC protein self-assemble to form a single flagellar filament [25], but despite their length, they form extremely stiff structures with an elastic modulus estimated to be over 10^10^ Nm^−2^ [26]. In addition, these filaments remain stable at temperatures up to 60 °C and under relatively acidic or basic conditions [27,28]. It is this durability that makes flagella-based nanofibers of particular interest for applications that require harsh environmental conditions.

Initial adaptation of the flagellar system for bionano applications targeted *E. coli* flagellin, where thioredoxin (*trxA*) was internally fused into the *fliC* gene, resulting in the FliTrx fusion protein [29]. This fusion resulted in a partial substitution of the flagellin D2 and D3 domains, with TrxA being bounded by G243 and A352 of FliC, importantly keeping the TrxA active site solvent accessible. The exposed TrxA active site was then used to introduce genetically encoded peptides, including a designed polycysteine loop, to the FliTrx construct. Since the domains responsible for self-assembly remained unmodified, flagellin nanotubes formed having 11 flagellin subunits per helical turn with each unit having the ability to form up to six disulfide bonds with neighboring flagella in oxidative conditions. Flagella bundles formed from these Cys-loop variants are 4−10 µm in length as observed by fluorescence microscopy and represent a novel nanomaterial. These bundles can be used as a cross-linking building block to be combined with other FliTrx variants with specific molecular recognition capabilities [29]. Other surface modifications of the FliTrx protein are possible by the insertion of amino acids with preferred functional groups into the thioredoxin active site. Follow-up studies by the same group revealed a layer-by-layer assembly of streptavidin-FliTrx with introduced arginine-lysine loops producing a more uniform assembly on gold-coated mica surfaces [30].

**Figure 1:**
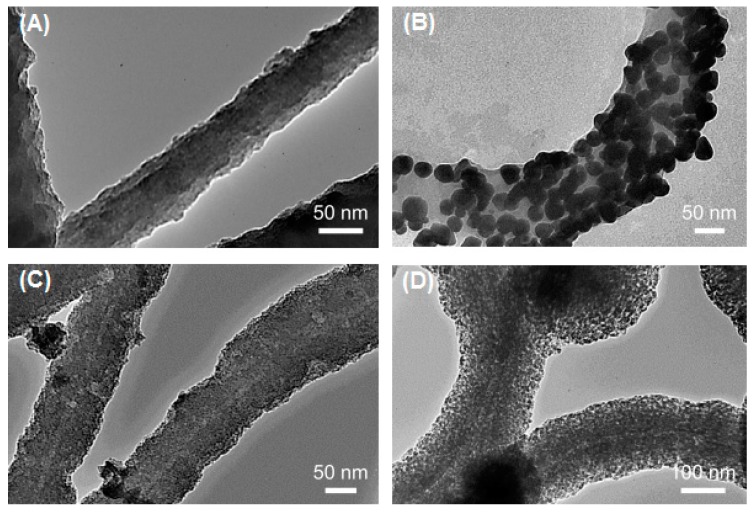
Transmission electron microscope (TEM) micrographs of pristine and metalized Flagella-templated silica nanotubes. (**A**) Pristine silica nanotubes fabricated on flagella bio-templates. (**B**) Gold, (**C**) palladium, and (**D**) iron oxide nanoparticles deposited on the silica nanotubes. (Reprinted with permission from Jo et al. *Nanotechnology* **24**, 13574 (2013) [33]).

Flagellin is increasingly being explored as a biological scaffold for the generation of metal nanowires. Kumara et al. [31] engineered the FliTrx flagella with constrained peptide loops containing imidazole groups (histidine), cationic amine and guanido groups (arginine and lysine), and anionic carboxylic acid groups (glutamic and aspartic acid). It was found that introduction of these peptide loops in the D3 domain yields an extremely uniform and evenly spaced array of binding sites for metal ions. Various metal ions were bound to suitable peptide loops followed by controlled reduction. These nanowires have the potential to be used in nanoelectronics, biosensors and as catalysts [31]. More recently, unmodified *S. typhimurium* flagella was used as a bio-template for the production of silica-mineralized nanotubes. The process reported by Jo and colleagues in 2012 [32] involves the pre-treatment of flagella with aminopropyltriethoxysilane (APTES) absorbed through hydrogen bonding and electrostatic interaction between the amino group of APTES and the functional groups of the amino acids on the outer surface. This step is followed by hydrolysis and condensation of tetraethoxysilane (TEOS) producing nucleating sites for silica growth. By simply modifying reaction times and conditions, the researchers were able to control the thickness of silica around the flagella [32]. These silica nanotubes were then modified by coating metal or metal oxide nanoparticles (gold, palladium and iron oxide) on their outer surface (Figure 1). It was observed that the electrical conductivity of the flagella-templated nanotubes improved [33], and these structures are currently being investigated for use in high-performance micro/nanoelectronics.

### 2.2. Pilin-Based Protein Nanotubes

**Figure 2:**
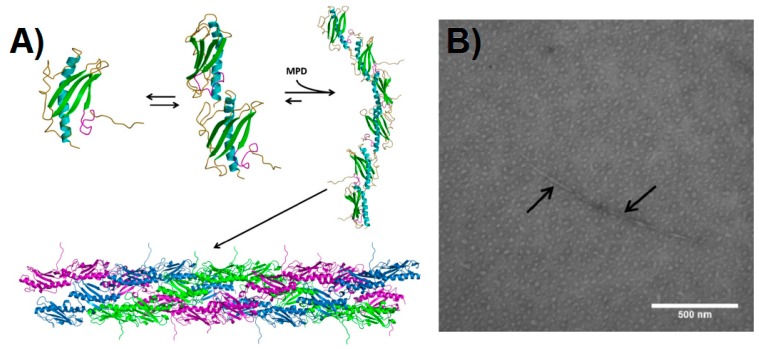
Pilin-derived protein nanotube (PNT) assembly. (**A**) The ΔK122 pilin (PBD ID 1QVE [45]) exists as a monomer-dimer equilibrium in solution [55]. The common structural features of the type IV pilins are highlighted in the monomer—the N-terminal α-helix in cyan, the β-sheet in green, coil regions in gold, and the receptor-binding domain (known to mediate surface interactions) in magenta. Upon addition of a hydrophobic compound such as 2-methyl-2,4-pentanediol (MPD), the ΔK122 pilin forms fibrils that can then assemble into PNTs. The three ΔK122 fibrils observed in a helical assembly of native T4P are shown in purple, green, and blue, respectively. (**B**) Upon the addition of the oligomerization initiator MPD, the ΔK122 monomer/dimers are seen as aggregates in TEM, and form pilin fibrils (highlighted by arrows). (Reprinted with permission from Petrov et al. *J. Nanobiotechnol*. **11**, 24 (2013) [54]).

Type 4 Pili (T4P) are polymers of a single monomeric type IV pilin subunit that extends from the surface of gram-negative bacteria to form fiber-like structures with a length ranging several micrometers and a diameter of approximately 6 nm [34,35,36]. Bacteria utilize T4P to mediate a variety of biological processes including cell-host attachment, microcolonization, biofilm formation, and twitching motility [37,38,39,40,41]. Atomic models for pilins from several bacteria have been characterized including, among others, pilins from *P. aeruginosa* strains PAK [42,43], K122-4 [44,45], PAO [46], Pa110594 [47], *Neisseria gonorrhoeae* strain MS11 [48], *Clostridium difficile* [49,50], and the toxin coregulated pilin (TcpA) of *Vibrio cholerae* [36]. Pilin proteins are comprised of a long N-terminal α-helix, a four-stranded antiparallel β-sheet with connecting loops, and a C-terminal disulfide bounded receptor-binding D-region [15]. The assembly of T4P has been well studied; all T4P models place the hydrophobic N-terminal α-helix in the interior of the pilus while the variable β-sheets are exposed on the outer surface [51]. Thus, the N-terminal α-helix is protected from the immune system and acts as a conserved oligomerization domain [8,15,45]. Recent work on the K122-4 pilin from *P. aeruginosa* has revealed that the protein oligomerizes into nanotubes in the presence of hydrophobic surfaces or compounds (Figure 2) [52,53,54,55]. While generated in vitro, the pilin-derived PNTs share a similar morphology and diameter (~5–6 nm) to in vivo T4P [52,53,54], the former can reach a length of several hundred micrometers compared to native pili that typically have a length of 10 µm [34,35,36,51].

From a bionanotechnology perspective, T4P form robust nanofibers with the ability to bind biotic and abiotic surfaces via their tips. These interactions have been mapped to the D-region of the pilin. It has been estimated that the attractive force between the native T4P tip and steel is in the range of 26–55 pN/molecular interaction and for in vitro derived nanotubes is in the range of 78–165 pN/molecular interaction [56]. Functional nanostructures have been generated from native bacterial pili and explored for their potential use as biological nanowires. For example, the type IV pili of *Geobacter sulfurreducens* reduces Fe(III) oxides by transporting electrons over long distances and has potential applications for use in microbial-based fuel cells [57,58]. Further studies have shown that cultures of *G. sulfurreducens* produce biofilms that exhibit high current densities—one of the highest known current densities when incorporated into microbial fuel cells [59]. These *G. sulfurreducens* pili are capable of long-range metallic-like conductivity [60] and supercapacitor behavior [61], making them an exciting prospect for use as a low-cost and environmentally sustainable form of energy storage.

The β-sheet and connecting loops of the type IV pilins form the surface of the pilus, and are thus exposed to the immune system. As a result these regions show significant sequence variability between bacterial systems. This allows for the use of mutagenesis to design fibers with altered surface properties. Research is ongoing to explore how protein engineering of the monomer can lead to nanofiber attachment to other abiotic surfaces. For instance, addition of a polyhistidine tag to the C-terminus of the protein can potentially direct binding to nickel and copper surfaces or nanoparticles. If we consider binding of T4P/PNT to biotic surfaces such as epithelial cells, this opens an exciting area for further research in therapeutics. As is the case with binding to abiotic surfaces, the D-region of the pilin is responsible for forming specific interactions with cellular glycolipids [62]. This receptor-specific interaction can allow for mediated drug delivery upon binding of the synthetic nanofibers.

## 3. Virus-Based Protein Nanotubes (PNTs)

Viral capsids are protein shells that serve to protect the enclosed genetic material. These self-assembling capsids are formed from relatively simple protein building blocks making them ideal for the production of nanostructures. Capsids vary in size from 18–500 nm with morphologies ranging from helical (rod-shaped) to icosahedral (spherical-shaped). These structures can be chemically and genetically manipulated to fit the needs of various applications in biomedicine, including cell imaging and vaccine production, along with the development of light-harvesting systems and photovoltaic devices. Due to their low toxicity for human applications, bacteriophage and plant viruses have been the main subjects of research [63]. Below, we highlight three widely studied viruses in the field of bionanotechnology.

### 3.1. Tobacco Mosaic Virus (TMV)

The concept of using virus-based self-assembled structures for use in nanotechnology was perhaps first explored when Fraenkel-Conrat and Williams demonstrated that tobacco mosaic virus (TMV) could be reconstituted in vitro from its isolated protein and nucleic acid components [64]. TMV is a simple rod-shaped virus made up of identical monomer coat proteins that assemble around a single stranded RNA genome. RNA is bound between the grooves of each successive turn of the helix leaving a central cavity measuring 4 nm in diameter, with the virion having a diameter of 18 nm. It is an exceptionally stable plant virus that offers great promise for its application in nanosystems. Its remarkable stability allows the TMV capsid to withstand a broad range of environments with varying pH (pH 3.5–9) and temperatures up to 90 °C for several hours without affecting its overall structure [65]. Early work on this system revealed that polymerization of the TMV coat protein is a concentration-dependent endothermic reaction and depolymerizes at low concentrations or decreased temperatures. According to a recent study, heating the virus to 94 °C results in the formation of spherical nanoparticles with varying diameters, depending on protein concentration [66]. Use of TMV as biotemplates for the production of nanowires has also been explored through sensitization with Pd(II) followed by electroless deposition of either copper, zinc, nickel or cobalt within the 4 nm central channel of the particles [67,68]. These metallized TMV-templated particles are predicted to play an important role in the future of nanodevice wiring.

**Figure 3:**
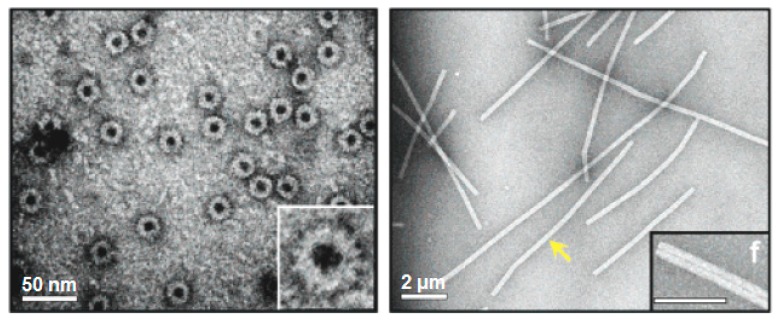
Viral protein-based nanodisks and nanotubes. TEM images of chromophore containing nanodisks (**left**) and nanotubes (**right**) produced from a modified tobacco mosaic virus (TMV) coat protein [69]. The scale bars represent 50 nm (**left**) and 200 nm (**right**). The yellow arrow is pointing to a single 900-nm-long TMV PNT containing over 6300 chromophore molecules. (Reprinted with permission from Miller et al. *J. Am. Chem. Soc*. **129**, 3104-3019 (2007) [69]).

Another interesting application of TMV has been in the creation of light-harvesting systems through self-assembly. Recombinant coat proteins were produced by attaching fluorescent chromophores to mutated cysteine residues. Under appropriate buffer conditions, self-assembly of the modified capsids took place forming disc and rod-shaped arrays of regularly spaced chromophores (Figure 3). Due to the stability of the coat protein scaffold coupled with optimal separation between each chromophore, this system offers efficient energy transfer with minimal energy loss by quenching. Analysis through fluorescence spectroscopy revealed that energy transfer was 90% efficient and occurs from multiple donor chromophores to a single receptor over a wide range of wavelengths [69]. A similar study used recombinant TMV coat protein to selectively incorporate either Zn-coordinated or free porphyrin derivatives within the capsid. These systems also demonstrated efficient light-harvesting and energy transfer capabilities [70]. It is hypothesized that these artificial light harvesting systems can be used for the construction of photovoltaic and photocatalytic devices.

### 3.2. Cowpea Mosaic Virus (CPMV)

The cowpea mosaic virus (CPMV) is approximately 30 nm in diameter with a capsid composed of 60 copies of both large (L, 41 kDa) and small (S, 24 kDa) proteins [71]. This icosahedral virus has coat proteins with exposed N- and C-termini allowing for peptides to be added onto the surface through genetic engineering. For example, virus-templated silica nanoparticles were produced through attachment of a short peptide on the surface exposed βB-βC loop of the S protein [72]. This site has been most frequently used for the insertion of foreign peptides between Ala22 and Pro23 [73]. CPMV has also been widely used in the field of nanomedicine through a variety of in vivo studies. For example, it was discovered that wild-type CPMV labelled with various fluorescent dyes are taken up by vascular endothelial cells allowing for intravital visualization of vasculature and blood flow in living mice and chick embryos [74]. Moreover, the intravital imaging of tumors continues to be challenging due to the low availability of specific and sensitive agents showing in vivo compatibility. Brunel and colleagues [75] used CPMV as a biosensor for the detection of tumor cells expressing vascular endothelial growth factor receptor-1 (VEGFR-1), which is expressed in a variety of cancer cells including breast cancers, gastric cancers, and schwannomas. Therefore, a VEGFR-1 specific F56f peptide and a fluorophore were chemically ligated to surface exposed lysines on CPMV. This multivalent CPMV nanoparticle was used to successfully recognize VEGFR-1-expressing tumor xenografts in mice [75]. In addition, use of the CPMV virus as a vaccine has been explored by the insertion of epitopes at the same surface exposed βB-βC loop of the small protein capsid mentioned earlier. One group found that insertion of a peptide derived from the VP2 coat protein of canine parvovirus (CPV) into the small CPMV capsid was able to confer protection in dogs vaccinated with the recombinant plant virus. It was found that all immunized dogs successfully produced increased amounts of antibodies specific to VP2 recognition [76].

### 3.3. M13 Bacteriophage

The M13 bacteriophage is perhaps the most widely studied virus in terms of bionanotechnology and nanomedicine. The virion is approximately 6.5 nm in diameter and 950 nm in length enclosing a circular single-stranded DNA genome. The helical capsid is composed of approximately 2700 copies of the major pVIII coat protein and is capped with 5 copies each of the pIII, pVI, pVII, and pIX minor coat proteins [77]. The process of phage display, which utilizes the ease of genetic manipulation to modify the surface proteins the M13 phage [78], has enabled this simple phage to be used for multiple purposes including peptide mapping [79], antigen presentation [80,81], as well as a therapeutic carrier and bioconjugation scaffold [82].

**Figure 4:**
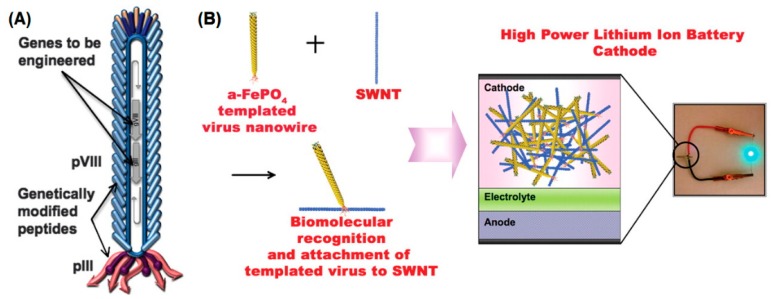
Genetically engineered M13 bacteriophage used as a lithium-ion battery cathode. (**A**) The gene VIII protein (pVIII), a major capsid protein of the virus, is modified to serve as a template for amorphous anhydrous iron phosphate (a-FePO_4_) growth. The gene III protein (pIII) is also engineered to have a binding affinity for single-walled nanotubes (SWNTs). (**B**) The fabrication of genetically engineered high-power lithium-ion battery cathodes and a photograph of the battery used to power a green light-emitting diode (LED). (Reprinted with permission from Lee et al. *Science* **324**, 1051–1055 (2009) [86]).

Recently, the major capsid protein of the M13 virus has been genetically engineered to display substrate binding peptides on the outer surface to selectively bind various conducting molecules [83]. For example, recombinant pIII and pVIII coat proteins were used to select for peptide motifs that facilitated the formation of gold nanowires. Through an affinity selection/ biopanning process, a strong gold binding motif on pVIII containing four serine residues was identified [77], a motif shown to have a high affinity for gold lattices [84]. A streptavidin-binding 12-mer peptide was also inserted into the pIII coat protein for localization at one end of the helical capsid. Incubation with pre-synthesized 5-nm gold nanoparticles produced an ordered arrangement of the particles along the virion surface. The resulting Au-plated nanowires reached dimensions of 10 nm in diameter and approximately 1 µm in length [77]. Similarly, Nam and colleagues developed negative electrodes for use in lithium ion batteries using highly ordered M13-templated gold-cobalt oxide nanowires [85]. To do this, the group engineered a modified pVIII coat protein containing four consecutive N-terminal glutamate residues to bind cobalt oxide (Co_3_O_4_) along with an additional gold-binding peptide motif. This hybrid clone expressing both Au- and Co_3_O_4_-specific peptides produced a nanowire consisting of a small amount of Au nanoparticles combined with Co_3_O_4_. The hybrid nanowire was observed to improve initial and reversible storage capacity by approximately 30% compared to pure Co_3_O_4_ nanowires when tested at the same current [85]. In a later study [86], the pVIII protein was bound to FePO_4_ while the pIII protein was modified with a peptide sequence facilitating the interaction with single-walled carbon nanotubes (SWCNTs). This brought together the benefits of biologically ordered nanowires with the robustness of carbon nanotubes to produce high-power lithium-ion battery-like cathodes (Figure 4) [86].

Similar to CPMV, the M13 bacteriophage has been explored for use in cancer cell imaging and targeted drug delivery. Chemical modification of reactive groups on the M13 bacteriophage allowed for the attachment of small fluorescent molecules along with folic acid along its surface. Folic acid binds to the folate receptor, which is overexpressed in several cancers, facilitating uptake by the cell through endocytosis. The study found that successful binding and uptake of the dually modified bacteriophage by human BK cancer cells, enabling a multi-modal imaging platform [87].

In addition, the M13 bacteriophage has been shown to penetrate the central nervous system (CNS), which has made it the focus of studies looking to deliver protein antibodies across the blood–brain barrier. The first example utilizing the M13 phage as a vehicle for transporting surface-displayed antibodies to the CNS was undertaken for the early detection of Alzheimer’s disease [88]. In Alzheimer’s, characterized by the formation of β amyloid peptide (AβP) plaques, early detection is critical to obtain maximum benefits from available treatments. While there are many methods to detect amyloid plaques in post-mortem brain tissue, an effective in vivo imaging method remains elusive. A β-amyloid antibody fragment for specific detection of plaques in transgenic mice was used while for construction of a single-chain variable fragment (scFv), variable regions of the heavy and light genes of parental anti-AβP IgM 508 antibody were used [73]. The resulting scFv-508F fragment was fused to the minor coat protein pIII and the recombinant phage successfully delivered phage-displayed anti-β-amyloid antibodies into the brains of mice via intranasal administration [88]. Subsequent studies performed with radiolabeled antibodies containing an isotope suitable for in vivo diagnostic imaging (e.g., ^123^I) suggests that this approach could allow for early detection of the disease [89]. Similar research has looked at using antibody-displaying bacteriophage constructs for the treatment of drug addictions such as cocaine [90]. Other protein-based approaches, such as the use of catalytic antibodies specific for the cleavage of cocaine, have not been successful in crossing the blood–brain barrier. Therefore, the pVIII coat protein containing a phage-displayed murine monoclonal antibody termed GNC 92H2 with high affinity and specificity for cocaine were assembled and administered to rats with no observed physical side effects. Enzyme-linked immunosorbent assay (ELISA) analysis of rat serum from vaccinated subjects showed no appreciable production of antibodies to the phage, demonstrating that an immune response was not occurring [90]. These studies reveal that recombinant M13 bacteriophage offers a unique strategy to introduce therapeutic protein agents directly to the CNS.

## 4. Self-Assembling PNTs

While the study of existing natural structures is beneficial because their mechanism of assembly has been shaped by evolution, the dimensions of these nanotubes are more or less fixed and might not be able to adapt to the exact requirements essential for certain applications. For instance, flagella and pili lack an inner cavity available for chemical modification or packaging of active pharmaceutical ingredients (APIs) for drug delivery, although this can be modified (see Section 2.2). There are several well-known examples of self-assembling PNTs generated from stacked multimer rings. These systems generally allow for a greater control over the position of the modifications made on both the outer and inner surfaces of the PNT. Below, we summarize some well-known and promising examples of multimer proteins that have been the focus of recent studies.

### 4.1. The *trp* RNA Binding Attenuation Protein (TRAP) Nanotube

**Figure 5:**
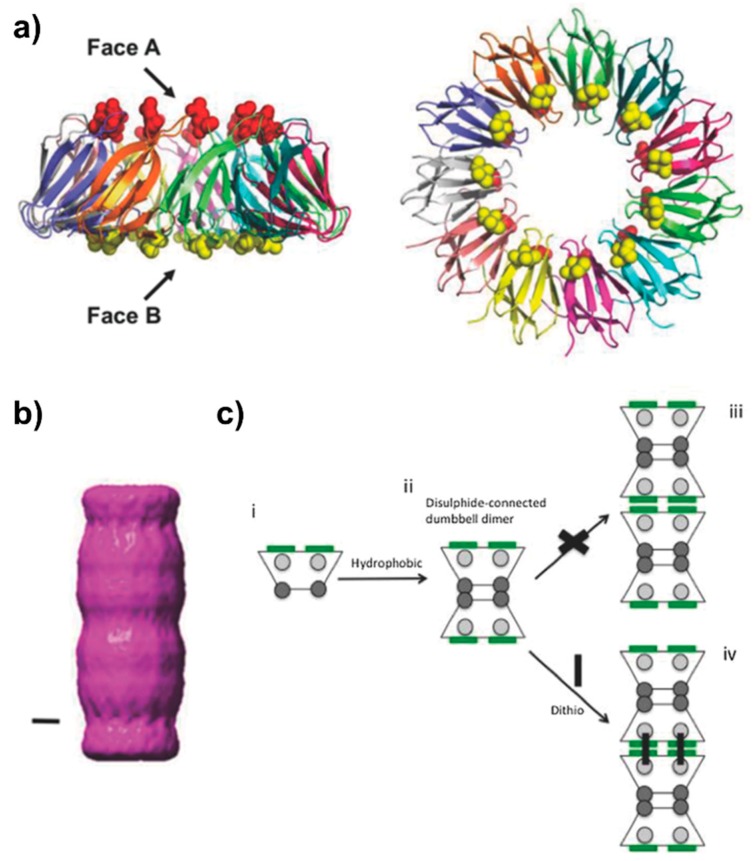
Design and assembly of PNTs of a mutant form of *trp* RNA-binding attenuation protein (TRAP) from *G. stearothermophilus*. (**a**) Side-on (left) and top-down (right) views of TRAP (PDB ID 1QAW [91]), colored by chain. The narrower “A” face harbors residue 50 (red sphere), while the wider “B” face harbours residue 69 (yellow sphere). In the original description of the TRAP PNTs [16], residues L50 and C69 allow for a hydrophobic-mediated interaction of the narrow “A” faces, and a dithio-mediated (such as via dithiothreitol, DTT) interaction of the “B” faces due to the steric bulk surrounding C69. (**b**) S Single particle analysis of the initial PNT forming “Tube TRAP” (TT) (scale bar represents 2 nm) [16], which was further modified to generate longer, more stable PNTs [18]. (**c**) Mutation L50C generates a di-cysteine mutant (TT_CC_) resulting in a much more stable PNT. Mechanistically, C50 on the narrow face (grey circles) can initially form direct disulfide bonds to form the initial TRAP dumbbell dimer; steric considerations prevent C69 interactions at this point. Addition of a dithio linker crosslinks the B faces via C69, resulting in an elongated TRAP PNT. Figure adapted with permission from Nagano et al. *Adv. Mater. Interfaces **3***, 1600846 (2016) [18].

The 8.2 kDa *trp* RNA binding attenuation protein (TRAP) from *Geobacillus stearothermophilus* forms an 11-mer thermostable ring that is 8.5 nm in diameter with a central cavity of approximately 2 nm [16]. Given its high stability, it is able to withstand various mutations while still maintaining its ring shape. Based on the crystal structure of the protein, mutants were designed in order to promote stacking of the TRAP rings into a tubular structure. To do this, cysteine residues were inserted at positions located on opposite faces of each monomer such that when two rings are brought together the cysteines align mediating the formation of disulfide bonds. Mutations V69C and E50L on the monomer place the cysteines approximately 2 nm from the center of the ring on each side, with a total of 11 cysteine resides per face (Figure 5). The mutant protein is able to assemble into nanotubes reaching up to 1 µm or more in length [16,18]. An additional mutant form L50C was optimized for ideal packing of the shorter face of the ring, termed Face A, forming a tightly packed dumbbell structure stabilized by direct disulfide bonds (Figure 5). These dumbbell-shaped dimers are then able to form bridged disulfide bonds through C69 on their wide interface (Face B) when a double-ended dithio linker such as dithiothreitol (DTT) is in solution under oxidizing conditions. This enables the assembly of the dimers into a polymeric nanotube that have higher resistance to dissociation from dilution [18].

The residues located in the inner cavity of TRAP are largely non-conserved [16,91], which allows further manipulation to tailor the TRAP NTs for a given application. For instance, mutations can be made to facilitate binding to metal ions for the production of nanowires or to chelate heavy metal contaminants that can then be filtered out of a solution. TRAP subunits could also be mutated to lower the hydrophobicity of the outer surface and increase solubility of the nanotube after assembly. Additionally, sequestration of small molecules within the interior of the TRAP NT could provide functionality as a drug delivery vehicle. Lastly, the TRAP monomer has been shown to bind RNA [17] and, therefore, the TRAP NT has the potential to function as a redox-sensitive delivery platform for RNA biomedicines such as RNAi, although this remains to be explored in detail. 

### 4.2. Microcompartment Proteins PduA and PduB

**Figure 6:**
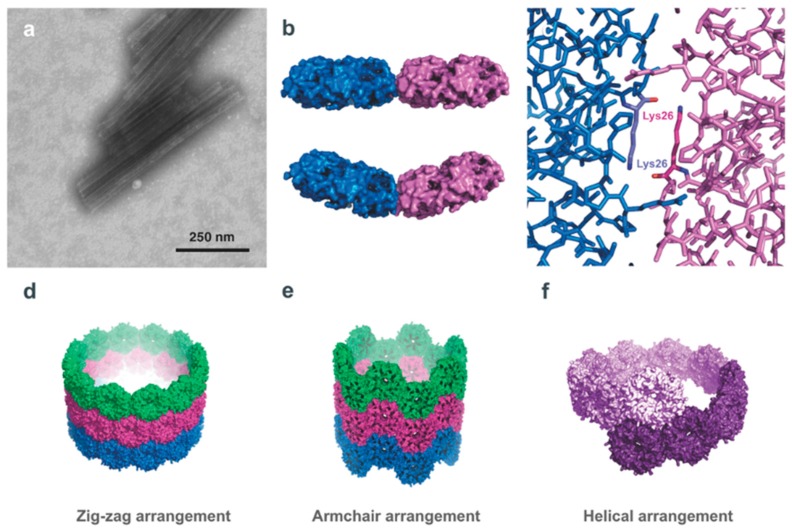
PNTs formed by the microcompartment protein PduA. (**a**) TEM image of PduA nanotubes, which indicate a consistent 20 nm diameter (lengths have been observed to differ). (**b**) A top-down view of two adjacent PduA hexamers (PDB ID 3NGK [95]) illustrating the hexamer–hexamer interface, at bend angles of 0° (top) and 36° (bottom). (**c**) Close up view of the PduA hexamer interface, highlighting the antiparallel arrangement adjacent Lys26 residues, held together by hydrogen bonding. It is this interaction that is critical for PNT assembly. (**d**–**f**) The three models of PduA PNTs: zig-zag, armchair, and helical, respectively. All three models result in a consistent 20 nm PNT diameter, though modelling suggests that the zig-zag or helical models of PduA PNT assembly more likely than an armchair assembly. All models present the convex face of the PduA hexamer, and importantly the N-terminus of the PduA monomer, to the exterior surface; this can allow the protein engineering of the N-terminus of the protein for surface display of a variety of moieties. (Figure adapted from Uddin et al. *Small* **14**, 1704020 (2018) [21], under the Creative Commons Attribution Licence).

A protein component of the *S. enterica* propanediol-utilization (Pdu) microcompartment shell, PduA, has been shown to spontaneously assemble into synthetic nanotubes with a diameter of 20 nm [21]. PduA assembles in vivo with seven other shell proteins that encapsulate an enzymatic core forming a closed pleomorphic organelle 100–150 nm in diameter [92,93,94]. When isolated, PduA forms bent hexamers with concave and convex faces that have been shown to form nanotubes that stack in predicted models at low salt (< 50 mM) concentrations (Figure 6) [21]. These model PNTs include (1) a zigzag form with 12 hexamers per turn where the flat edge of each hexamer is almost parallel to the tube’s axis causing a bend angle of 30°, (2) an analogous single-start helical model with 10 hexamers per turn, a 37.5° bend angle with an upwards pitch of 61 nm, and (3) a less favourable armchair model. The predicted models preserve the interaction of crucial hydrogen bonding between an antiparallel lysine pair seen in crystal structures and determined to be essential for PNT formation, and display the concave face of the PduA hexamers as exterior-facing. The N-terminus of the subunits in each hexamer was determined to be on the concave face, therefore the exterior of the PNT, allowing for modifications to be made to the PduA monomer that would enable scaffolding of enzymes or nanobodies to the surface of the filament [21]. Additionally, if biologically active molecules are desired to be sequestered in the lumen of the PduA nanotube then the convex face can be appropriately engineered.

**Figure 7:**
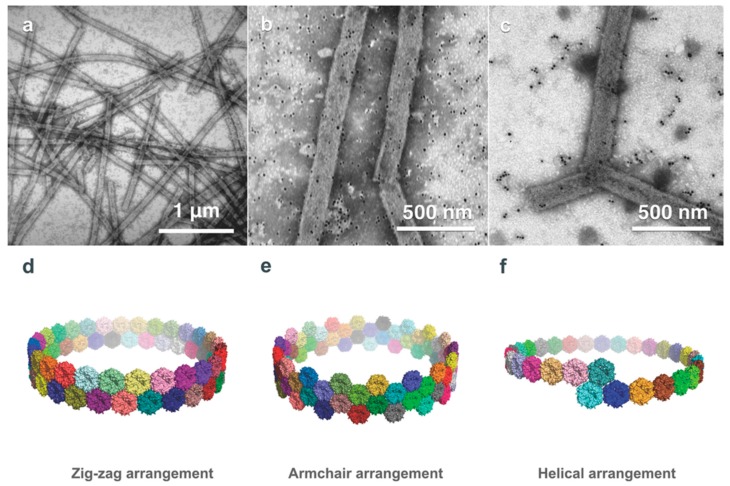
PduB-based PNTs. (**a**) TEM images of PduB PNTs indicate more structural diversity in length, diameter, and surface curvature than PduA-based PNTs. Labelling of His-tagged PduB PNTs with gold nanoparticles in 80 mM (**b**) and 250 mM (**c**) imidazole buffer, respectively, demonstrates that the concave surface of the PduB pseudo-hexamer faces the exterior of the PNT, enabling nanoparticle binding. Similar to PduA, PduB PNTs can form via zig-zag (**d**), armchair (**e**), and helical (**f**) arrangements; as with PduA, the PduB PNTs likely form via a zig-zag or helical arrangement. (Figure adapted from Uddin et al. *Small* **14**, 1704020 (2018) [21], under the Creative Commons Attribution Licence).

A trimeric microcompartment shell component protein PduB from *L. reuteri* forms psuedo-hexamers can also spontaneously form PNTs with a diameter of approximately 63 nm when isolated and dialyzed into low salt conditions [21]. These PNTs are much larger than PduA nanotubes and show more structural diversity (Figure 7), largely due to their shallower bend angle of the hexamers at the edge interface in which the antiparallel lysine interaction seen in PduA is not required for sufficient electrostatic bonding. The shape of the PduB hexamers is similarly bent such that the concave face is external and the convex face is lumen-facing; however, the N-terminus of each subunit lies internally in the PduB PNT. Modeling of the PduB hexamers into nanotubes shows similar favourable stacking patterns of the PduA nanotube; a zigzag model, an armchair model and a single-start helical model. These PduA and PduB nanotubes reveal a generic assembly process in spontaneous PNT formation and provide further options to those that may wish to engineer PNTs with targeted internal or external functionalities for biotechnology or biomedical applications.

### 4.3. Hcp1 Nanotubes

Hcp1 is a ring-shaped hexameric protein from *P. aeruginosa* and constitutes part of a type IV secretion system [19]. X-ray structure analysis revealed that Hcp1 consists of an outer diameter of 9 nm with an inner diameter of 4 nm and a height of 4.4 nm. Like TRAP, Hcp1 was modified to display cysteine residues (at G90 and R157) to produce engineered disulfide bonds on both faces of the ring. These disulfide bonds serve to stabilize the ring-ring interface and promote stacking of the hexamers into tubular structures. Under the right ionic and solvent conditions, PNTs containing 25 subunits corresponding to approximately 100 nm in length were formed. It was found that Hcp1 tube formation could be terminated with the addition of single-cysteine mutants. By varying the concentration of these chain-terminating subunits relative to the double-mutants capable of chain extension, one could control the extent of polymerization and length of the nanotubes [19].

### 4.4. Stable Protein 1 (SP1) Nanotubes

Stable protein 1 (SP1) is a stress response protein originally isolated from aspen (*Populus tremula*). The quaternary assembly of SP1 is a dodecameric ring measuring 11 nm in diameter with an inner pore of 2-3 nm and a height of 4–5 nm [96]. The SP1 ring is highly stable, with the N-terminus of the SP1 monomer located within the inner cavity of the ring; N-terminally modified SP1 is still capable of forming the quaternary ring assembly. Initial studies of the SP1 assembly highlighted that an N-terminal histidine tag enabled the bridging of gold nanoparticles on the inner surface of the SP1 rings [20], suggesting the potential for energy transfer applications. Incubation of the SP1 protein with CdTe quantum dots (QDs) of various sizes resulted in PNT-like structures that were both interior bound and inter-ring bound QDs [97]. These SP1-QD assemblies demonstrated Förster resonance energy transfer (FRET), indicating that these structures could efficiently transfer energy along their lengths and demonstrating the potential for bionano-based light harvesting or energy transfer applications [97]. Engineering of the SP1 monomer to include the active center of the antioxidant selenoenzyme glutathione peroxidase (GPx) on the inner surface of the SP1 ring resulted in a highly stable and enzymatically active SP1 chimera [98]. Subsequent studies of this chimeric SP1 monomer [99] produced highly ordered PNTs based on the zero-length cross-linking of SP1 rings using ethylenediamine (EDA) resulted in SP1 PNTs with increased GPx activity and increased stability profiles [99]. The SP1 platform is demonstrating to be a versatile and promising platform for multiple bionano-inspired applications.

### 4.5. Self-Assembling Protein Nanoparticle (SAPN) Malaria Vaccine

A new avenue of research looks at self-assembling PNTs that can be used for the development of vaccines against various diseases. For example, efforts are on-going to develop a vaccine against *Plasmodium falciparum* malaria using the circumsporozoite protein (CSP). CSP is a cell surface protein of the sporozoite, the stage in the life cycle of the malaria parasite that infects vertebrate hosts [100]. Recently, a chimeric *P. falciparum* protein has been designed that self-assembles into a repetitive antigen containing 60 units forming spherical particles of approximately 40 nm [101]. This protein contains B-cell, CD4^+^ T-cell, and three different CD8^+^ T-cell epitopes of the circumsporozoite protein of *P. falciparum* (PfCSP), as well as coiled-coil pentamer and trimer domains. Upon formation of the protein nanoparticle, the B-cell and CD8^+^ T-cell epitopes are exposed on the outer surface and CD4^+^ T-cell epitope is located in the internal core. The nanoparticle was able to trigger a humoral immune response in mice, protecting them from lethal doses of parasites expressing wild-type *P. falciparum* CSP [101].

### 4.6. Bacterial Gas Vesicles

The buoyancy of bacteria and archaea in aquatic environments is regulated through intracellular multi-protein complexes known as gas vesicles (GV) [102]. GVs are nanostructures (45−250 nm in width, 100−800 nm in length, 2 nm thickness) that are able to withstand exterior hydrostatic pressures while permitting the free diffusion of gases across the proteinaceous surface. GVs are composed of two primary structural proteins, gas vesicle proteins A (GvpA) and C (GvpC); the former an amphiphilic 7.4 kDa protein that self assembles into the shell and the latter binds to the GvpC-assembly surface and strengthens the vesicle [102]. There are also several other putative minor components and chaperones; in total GVs are encoded by nine different genes. The GV is a cone-shaped protein nanostructure that has high sensitivity of detection across a range of ultrasound frequencies at picomolar concentrations, exhibiting harmonic scattering for in vivo detection above background noise, and enabling multiplexed imaging due to species-dependent thresholds for pressure-induced collapse [103]. It is these properties that make them attractive as acoustic detectors for targeted ultrasound imaging [102,104].

GVs are self-assembling protein nanostructures that can be engineered to tailor their surface characteristics. GvpC has been shown to be a target for modification of GV surface properties including zeta potential, the display of ligands for reduced or enhanced cellular targeting and uptake, and the attachment of fluorescent proteins to enable multimodal imaging [104]. In assembled GVs, recombinant GvpC was shown to alter acoustic stability for multimodal imaging, modulation of harmonic ultrasound signals, and vesicle surface properties for cell-specific binding and fluorescent reporting [104]. Ultrasound imaging is an inexpensive, safe and non-invasive method compared to magnetic resonance or nuclear imaging. Through the engineering of the GvpC subunit, GVs have the potential to become an innovative and important target-specific ultrasound imaging agent.

## 5. Eukaryotic Systems

### 5.1. Kinesin-Microtubule Based Systems

Microtubules, along with actin filaments, make up part of the cytoskeleton giving cells their characteristic shape. They have an outer diameter of 25 nm, an inner diameter of 15 nm, and range up to several micrometers in length. They are composed of repeating α- and β-tubulin units and are largely responsible for the movement of vesicles, organelles and other substances within cells [105]. They are also involved in the very dynamic process of cell division. Depending on the requirements of the cell, microtubules are continuously assembled and disassembled. Therefore, it is no surprise that these structures are intrinsically unstable with polymerization depending on factors such as pH, temperature, and solvent.

Several examples of self-assembling biomolecular templates for use as nanowires have been discussed above. This has also been explored in the case of microtubules and metallization with copper. Copper is the most widely used metal in the production of conducting wires due to its high availability and low electrical resistance; a method has been recently reported for the production of copper coated microtubules using electroless copper deposition chemistry [105]. Briefly, this involves the addition of polymerized microtubules to a bath of copper sulfate solution containing acetic and ascorbic acids acid as the complexant and ascorbic acid as the reducing agent. Metallization appears to initiate from the inner core of the microtubules which contains a histidine site with the highest binding affinity for copper ions and after one minute produces nanowires with an average diameter of 15 nm [105].

### 5.2. Amyloid Fibrils

Protein polymers that lack a central cavity are also an interesting target for protein-based nanosystems development. One of the most well-known protein polymer/aggregates is the β-amyloid protein and associated fibrils that form neurodegenerative plaques that are the hallmark of Alzheimer’s Disease [106]. Similarly, the prion protein affects neurodegeneration via a misfolding event that results in the formation of filamentous neurodegenerative aggregates. In theory, these protein polymers can be linked to conducting materials forming nanowires for photovoltaic devices. For instance, the self-assembly of the N-terminal and middle region of Sup35p, a prion originating in yeast, produced amyloid fibers that could be coated with gold or silver, resulting in protein-based wires that were approximately 100 nm wide [107]; the gold/silver atoms were attached covalently to the external surface of the protein using a genetically modified variant containing accessible cysteine residues. However, the study found that it was difficult to control the length and shape of the wire, rendering applications to real-life systems questionable [107]. It is clear that further research is required to lend support to the proposed roles that amyloids can play in the field of nanotechnology such as in protein scaffolding, organic solar cells and nanowires.

### 5.3. Silk Proteins Sericin and Fibroin

The silk protein sericin is a serine rich, mainly β-sheet protein found in silks from arthropods [108]. The other major component of silk, the glycoprotein fibroin, makes up to 70% of secreted silks and is sheathed by sericin to form large macroscopic silk fibers [109]. Silk fibroin is used for the production of textiles due to its high dexterity, durability and light weight. As a by-product of commercial silk production from silkworms, leftover sericin from *Bombyx mori* has been extracted and recycled as a biomaterial since 1983 (reviewed in [110]).

There have been many developments relevant to the field of biomedicine involving the engineering of biomaterials from self-assembling silk protein nanostructures. The most commonly studied silk proteins, sericin and fibroin, are extracted from the cocoons of silk worm species *B. mori* [109], *Antheraea mylitta* [108], *Antheraea assamensis*, as well as fibroin proteins ADF3 and ADF4 from the spider *Araneus diadematu* [111]. *B. mori* sericin has been previously used in the production of films for enzyme immobilization [112,113]. The coating of GOx onto non-woven fibroin fabrics using aqueous solutions of sericin and/or fibroin provided a biocatalytic surface increasing protein stability and allowing for extended enzymatic activity, something potentially useful in the large-scale production of topically applicable pharmaceuticals. For instance fibroin, sericin, and composites containing both silk proteins have been used for wound dressings that aid in accelerated wound healing [110,113,114].

Engineered recombinant spider silk proteins ADF3 and ADF4 have proven to be adaptable through manipulation of liquid–solid phase transitions, leading to different materials morphologies with adjustable properties [111]. Reported materials include sphere- and capsule-like carriers useful in drug delivery, and a variety of film morphologies with biomedical applications in tissue engineering through their utility as a cell scaffold. While no silk PNT morphologies have been as yet identified, the function of these materials are notable for their biomedical applications. Silk from spiders of the genus *Nephila* has been investigated in the development of artificial nerve conduits that promote proper axonal regeneration [115], as well as in the formation of a biodegradable scaffold that provides the mechanical strength required for the reconstruction of a human bladder [116].

These silk structures could be adapted and improved through substitution with self-assembling silk-elastin-like protein polymers (SELPs); a genetically engineered protein block copolymer [117]. These structures consist of tandemly repeated units of silk-like (GAGAGS) and elastin-like (GXGVP) peptide blocks. The silk-like block sequence is adopted from the *B. mori* fibroin heavy chain, which assembles into β-sheets, essentially amyloids, thus providing the physical crosslinking for the polymeric system. The elastin-like block provides coacervation; where X in the sequence is any amino acid except for proline, which allows for a reversible response to external stimuli that can be tuned based on the X residue in elastin, the silk-elastin ratio, and the molecular weight of the protein (as dictated by the number of blocks in a single chain). SELPs have been used in the formation of nanoparticles for the delivery of drugs, including doxorubicin (DOX), and can be tuned to spontaneously self-assemble into sheets for the formation of cell scaffolds for tissue engineering and biosensors for reporter assays [118,119]. However due to their tunable properties they have the potential to be modified to serve any of the applications described for silk protein fibers.

### 5.4. Human Insulin-Like Growth Factor Binding Protein-2 (hIGFBP-2)

**Figure 8:**
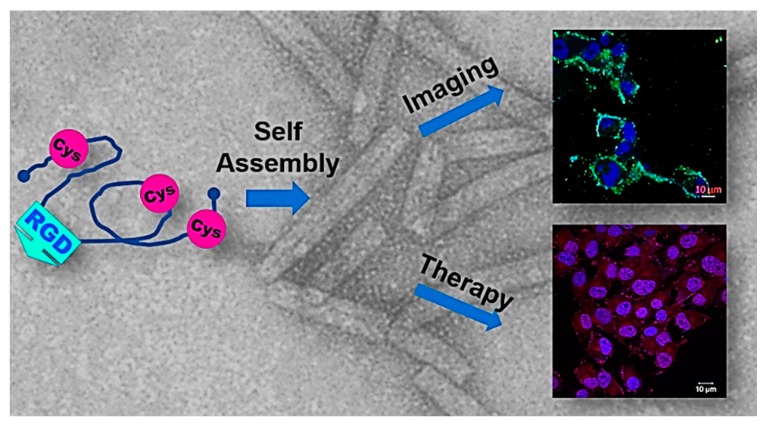
Multifunctional PNTs assembled from an engineered polypeptide from the human insulin-like growth factor binding protein-2 (hIGFBP-2). The polypeptide is generated from the C-terminal loop region (residues 249-289) of hIGFBP-2, with an additional cysteine added at position 281 (R281C) [127,128]. The presence of the three Cys residues (C249, C270, C281) results in the redox-controlled assembly/disassembly of PNTs. The recombinant hIGFBP-2 contains an RGD tripeptide enabling cellular targeting, and the PNTs can be labelled with FITC for imaging purposes or Doxirubicin for therapeutic action versus cancer cells. This multifunctional nature of the hIGFBP-2PNTs is an intriguing first step to adaptation of PNTs as multimodal targeting structures. (Figure adapted from Asampille et al. *J. Nanobiotechnol.* **16**, 101 (2018) [128] under the Creative Commons Attribution 4.0 License).

Another approach to generate eukaryotic protein nanotubes is to adapt a distinct domain or loop region of a protein precursor for PNT generation; this approach is unlike the use of synthetic peptides for PNT synthesis, of which there are numerous examples including [120,121,122,123,124,125,126], among many others. A recent example of the use of a protein’s loop region for PNT formation with potential therapeutic and imaging applications is the human insulin-like growth factor binding protein-2 (hIGFBP-2) [127,128]. In the structure of hIGFBP-2, the C-terminal region of the protein, C249-Q289, is largely unstructured and very dynamic [129]. This loop region also contains an RGD tripeptide (residues 265-267) [129]; RGD tripeptides are well known as a cellular targeting motif, primarily through integrin binding [130]. Examination of the hIGFBP-2_249-289_ polypeptide indicated that while the native sequence remained monomeric, addition of at third Cys residue at position 281 facilitated the self-assembly of the polypeptide into tubular structures [127,131] (Figure 8). Subsequent characterization of these hIGFBP-2 PNTs determined that self-assembly/disassembly is redox reversible, and labelling the hIGFBP-2 PNTs enabled cellular visualization [128]. Interestingly, the hIGFBP-2 PNTs could be loaded with DOX, and that these DOX-loaded PNTs could increase DOX uptake in cells for increased cytotoxicity in cancer cells. The RGD targeting and ability to load the hIGP PNTs opens interesting avenues for medical applications (see below), and the adaptation of other PNTs for similar applications.

## 6. Medical Applications of Protein and Peptide NTs

The utility of PNTs in medical applications, such as modification of viral capsids for drug delivery and disease detection, and the use of the protist CSP for malaria vaccination, has been briefly outlined above. However, many eukaryotic and prokaryotic proteins have been used to form PNTs with properties presenting opportunities for applications in the medical and food industries. As mentioned in Section 2.1, the FliC flagellar nanotube protein subunit from *E.coli*, has been used to form anionic PNTs that can coordinate cationic liposomes loaded with an anticancer drug zinc phthalocyanine (ZnPc) [132]. The FliC PNTs stabilize the liposome by reducing ZnPc vibrational motion, allowing for increased circulation time of the drug delivery system. Other drugs could conceivably be loaded into cationic liposomes and stabilized by these flagellar PNTs, thus providing the pharmaceutical industry a novel vehicle for drug delivery. Table 1 highlights the medical applications of PNTs discussed in this article, including PNTs from globular proteins that are cleaved to become self-assembling polypeptides.

Amelogenin is an extracellular matrix protein found in developing dental enamel [133]. Ameloblasts secrete amelogenin, which is then proteolytically degraded into shorter segments that self-assemble to form nanospheres that can organize into supramolecular rod structures upon which hydroxylapatite crystals form, creating the hardest tissue in vertebrates, mature enamel [134]. Amelogenin nanorods, an intermediate structure of enamel formation, have been achieved through controlled self-assembly of amelogenin nanospheres from recombinant full-length amelogenin upon which calcium phosphate nucleates to form nanocrystallites [133]. These nanorods can be further assembled to form hierarchically organized microstructures of similar physical and morphological characteristics as mature enamel. Another study by Wang et al. [135] showed that hierarchically organized nanorod structures can be induced through a constant composition method in which physiological conditions for in vivo enamel growth are mimicked. The strength of this biomaterial provides many opportunities for use in dentistry, controlled self-assembly of amelogenin nanorods into an enamel-like biomaterial has been suggested for repair of dental caries and enamel.

Another globular eukaryotic polypeptide that self-assembles to form a PNT is the proteolytically cleaved product of α-lactalbumin, a milk protein typically studied from bovine sources with a native function of lactose production in mammary glands. When partially hydrolyzed by a protease from *Bacillus licheniformis*, the resultant 10–14kDa molecules self-assemble to form nanotubes with unique properties [136,137]. Tubular structures are reproducible with strict minimum concentrations of protein and a divalent cation, optimally Ca^2+^ [136]. Lowering of calcium ion concentration through dilution causes a controlled disassembly at a rate linearly proportional to the inverse Ca^2+^ concentration. Despite the variety of cleavage products, α-lactalbumin nanotubes are regular structures that are typically longer than 100 nm and have a diameter of 20 nm with an 8 nm hollow core [138].

Nanotubes from α-lactalbumin are of particular interest to the food and pharmaceutical industries as products made by these PNTs will be easily accepted by the US Food and Drug Administration (FDA); α-lactalbumin is found at 3.5% in pasteurized bovine milk, and human α-lactalbumin is the principal protein of human milk [136]. Advantageous properties include the stability of the nanotubules; they can withstand treatments common in industrial manufacturing such as pasteurization and freeze drying. The mechanical strength of α-lactalbumin nanotubes is superior to myofibrils or casein micelles but slightly softer than microtubules or viral capsids, however the linearity of the α-lactalbumin nanotubes allows for their stacking to form a strong gel [137]. The controllable disassembly of this gel through lowering of Ca^2+^ concentration makes α-lactalbumin PNTs suitable as gelation agents or viscosifiers in food products with the ability to controllably reduce gel strength. As well the presence of a core allows for encapsulation of chemicals, providing potential as a drug delivery vehicle or for nutraceuticals; dietary compounds with medical benefits that typically have low intestinal absorption [139].

Lastly, the RGD-containing hIGFBP-2 PNTs (Section 5.4) [128] present an intriguing avenue for cancer treatment using PNTs. Not only are these PNTs themselves capable of cellular imaging and drug delivery, they suggest that engineering of other PNT precursors, for instance type IV pilins, could be a promising avenue for developing multiple PNT solutions for nanomedical applications.

## 7. Conclusions and Future Perspectives

PNTs are an analogous platform to inorganic (carbon- or silica-based) nanotubes, however they exhibit less adverse properties (immunogenicity, toxicity etc.) in biological environments. There is a richness of diversity in protein-based structures available for use in the fields of bionanotechnology and nanomedicine. The in-built complexity of full-length proteins, coupled with the assembly propensities of naturally occurring structures such as flagella, pili, and viral coat proteins present interesting opportunities in the design and development of these bionanosystems. Additionally, genetic engineering of multimeric ring proteins could be engineered for enzymatic activity or nanoparticle binding. Future studies characterizing the assembly kinetics, enhancing stability, and tailoring loading capacities will facilitate the development of implantable nanoelectronics for medical biosensors and targeted biocompatible methods for the detection and treatment of disease.

## Figures and Tables

**Table 1:** Applications of PNTs in the medical, pharmaceutical and food industries.

PNT Origin	Application	Reference
**Bacterial**		
Flagella (FliC)	Anionic FliC PNTs coordinated with ZnPc-loaded cationic liposomes are used for targeted drug delivery of cancer cells	[132]
Gas vesicles	GvpC is engineered to alter surface properties, the display of ligands, and the attachment of fluorescent proteins to enable enhanced ultrasound imaging	[104]
**Viral**		
Cowpea mosaic virus (CPMV) capsid	Labeled with fluorescent dye for non-immunogenic intravital visualization of vasculature	[74]
	Labeled with a peptide and a fluorophore for tumor cell detection	[75]
	Attachment of viral antigens as a vaccine delivery vehicle	[76]
M13 Bacteriophage	Folic acid and fluorophore attachment allows visualization of cancer cells overexpressing the folate receptor	[87]
	Detection of Alzheimer’s through fusion of M13 minor coat protein pIII with I^123^ radiolabeled anti-β-amyloid antibody	[88,89]
	Attachment of GNC 92H2 murine monoclonal antibody to the pVIII coat protein for treatment of cocaine addictions	[90]
**Eukaryotic**		
Self-Assembled Protein Nanoparticle (SAPN)	Self-assembled spherical nanoparticles presenting *P. falciparum* antigenic epitopes for use as a malarial vaccine	[101]
α-lactalbumin	PNTs with utility as viscosifiers, gelation agents for food and encapsulating vitamins or enzymes for drug delivery	[136]
Amelogenin	Amelogenin nanorods facilitate enamel growth in dental repair	[135]
Human insulin-like growth factor binding protein-2 (hIGFPB-2)	Cell targeting with DOX and imaging agents	[127,128]
Silk Proteins	*B. mori* silk processed to immobilize enzymes for the production of a biocatalytic surface	[109]
	Adaptable ADF3 and ADF4 fibroin forms morphologies suitable as drug delivery vehicles, and for cell adhesion during tissue engineering	[111]
	*Nephila* spider silk is used as a biocompatible scaffold, shown to guide axonal regeneration and allow tissue development for the reconstruction of a human bladder	[115,116]
	Silk-elastin-like protein polymers tuned to form a nanoparticle capable of targeting cancer cells with doxorubicin	[119]
